# Management of human resources for health in health districts in Uganda: A decision space analysis

**DOI:** 10.1002/hpm.3359

**Published:** 2021-10-25

**Authors:** Wesam Mansour, Adelaine Aryaija‐Karemani, Tim Martineau, Justine Namakula, Paul Mubiri, Freddie Ssengooba, Joanna Raven

**Affiliations:** ^1^ Department of International Public Health Liverpool School of Tropical Medicine Liverpool UK; ^2^ Department of Health Policy Planning and Management Makerere University School of Public Health Kampala Uganda

**Keywords:** decentralisation, decision space, DHMT, district health managers, Uganda

## Abstract

**Background:**

Decentralisation has been adopted by many governments to strengthen national systems, including the health system. Decision space is used to describe the decision‐making power devolved to local government. Human resource Management (HRM) is a challenging area that District Health Management Teams (DHMT) need some control over its functions to develop innovative ways of improving health services. The study aims to examine the use of DHMTs' reported decision space for HRM functions in Uganda.

**Methods:**

Mixed methods approach was used to examine the DHMTs' reported decision space for HRM functions in three districts in Uganda, which included self‐assessment questionnaires and focus group discussions (FGDs).

**Results:**

The decision space available for the DHMTs varied across districts, with Bunyangabu and Ntoroko DHMTs reporting having more control than Kabarole. All DHMTs reported full control over the functions of performance management, monitoring policy implementation, forecasting staffing needs, staff deployment, and identifying capacity needs. However, they reported narrow decision space for developing job descriptions, resources mobilisation, and organising training; and no control over modifying staffing norms, setting salaries and developing an HR information system (HRIS). Nevertheless, DHMTs tried to overcome their limitations by adjusting HR policies locally, better utilising available resources and adapting the HRIS to local needs.

**Conclusions:**

Decentralisation provides a critical opportunity to strengthen HRM in low‐and‐middle‐income countries. Examining decision space for HRM functions can help identify areas where district health managers can change or improve their actions. In Uganda, decentralisation helped the DHMTs be more responsive to the local workforce needs and analysing decision space helped identify areas for improvement in HRM. There are some limitations and more power over HRM functions and strong management competencies would help them become more resourceful.

## 1 BACKGROUND

Decentralisation is a critical reform initiative that governments in high‐income countries, as well as, in low‐ and middle‐income countries (LMICs), commonly adopt to strengthen health system's functions and improve service delivery.^1^ The World Health Organization (WHO) identifies four key functions for the health system: stewardship; financing; creating resources; and provision of services.^2^ Decentralisation could provide different levels of control over these functions through various decision‐making mechanisms which mostly involve transferring authority to the local level.^3^, ^4^, ^5^, ^6^, ^7^, ^8^ Accordingly, the concept of ‘decision space’ was developed to describe the decision‐making power, authority, and accountability at the local level.^3^, ^9^, ^10^, ^11^, ^12^ Decision space is defined as ‘the range of effective choice that is allowed by central authorities (the principal) to be utilized by local authorities (the agents)’^1^ (p. 1518). Although, this range of choices is formally provided by law and local policies as ‘de jure’ decision space, local authorities might experience a different range of choices from that formally agreed, which is referred to as the actual or ‘de facto’ decision space.^9^ Decentralisation can increase the autonomy of managers and create more decision space. As reported by the World Bank (1993), transferring authority to local government is meant to improve allocative and technical competencies, and promote equity and responsiveness to local needs and quality of care.^13^ Similarly, Bossert claims that, in decentralised contexts, there may be varying levels of decentralisation in devolved structures that provide district managers with a certain level of autonomy or decision space.^1^

Human resources for health (HRH) is a challenging area of local decision space yet control over it is vital since it can have an impact on other functions in the health system.^3^ Developing local control over numbers, cadres and salaries of staff is a good step towards the transfer of decision space from the central to local level in some decentralised contexts, and having authority over the hiring, dismissal and supervision of staff can also endorse governance and support decision space.^3^ Bossert and Beauvais argue that centralisation of Human Resource Management (HRM) functions can significantly limit local decision space provided to managers in other health system areas such as financing and service delivery.^3^, ^7^ In decentralised contexts, increased decision space for managers for utilisation of allocated budgets according to their needs and to exercise the managerial authority over HRM functions would improve service delivery and promote community engagement.^7^, ^9^ For example, Bossert examined the incentives that central level offered to local governments to encourage them to achieve health objectives, and found that increasing flexibility on decisions about HRM functions—that allow for productivity and quality incentives for health facilities and allow managers greater ability to hire and dismiss—can increase efficiency and quality of care.^1^ In Ghana, the limited decision space in HRM at the subnational levels resulted in negative impact on the effectiveness of some HRM functions such as recruitment, retention, and deployment of staff.^14^ This level of autonomy offers district level managers such as the District Health Management Team (DHMT) the opportunity to move beyond its normal management (mainly administrative functions of planning, resourcing, monitoring, etc.) towards more entrepreneurial activities^15^ that can improve or even transform health systems in ways that will support the achievement of Universal Health Coverage (UHC). Some authors argue that UHC cannot be achieved without strong local (e.g., district) health systems.^16^ However, the transfer of management authority to the local levels should be accompanied by adequate decision‐making and management capacities for the health managers to perform better and improve health services.^17^, ^18^

## 2 THE STRUCTURE OF THE DECENTRALISED HEALTH SYSTEM IN UGANDA

**FIGURE 1:**
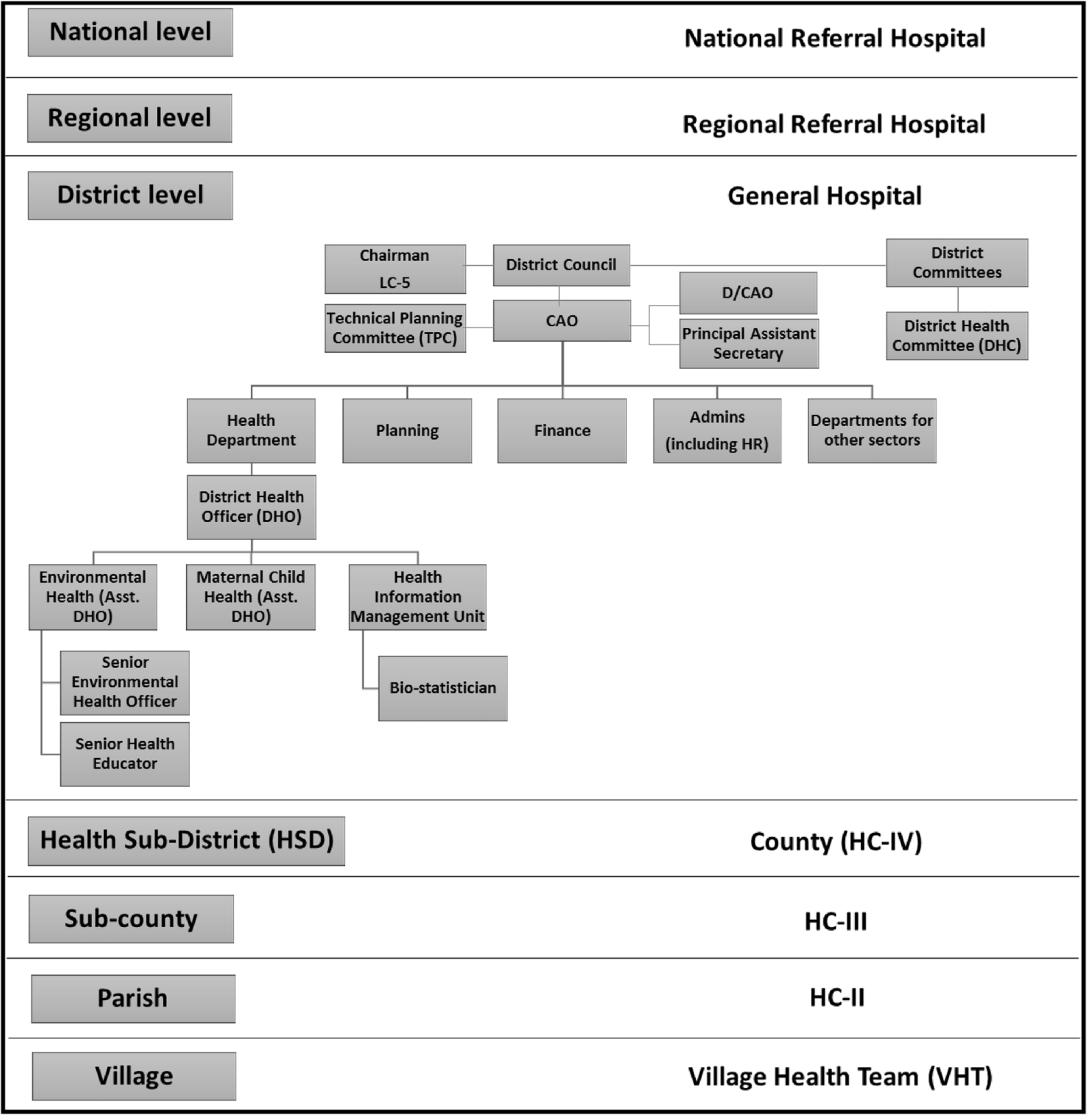
Structure of the decentralised health system in Uganda

Uganda began implementing its decentralisation policy in 1986 but administrative decentralisation did not start until the early 1990s^19^ (p. 18). The Ugandan government adopted a devolution mechanism in the health sector, transferring decision‐making capacities to local government authorities.^20^, ^21^ The government health service is structured into national, district and sub‐district levels (Figure 1).^22^, ^23^, ^24^ From the 1990s new districts have been created from existing districts^25^—referred to as “district splitting”—which has been quite rapid with the number of districts tripling between 1990 and 2016.^26^ The Health Sub District (HSD) acts as a functional subdivision of the district health system, connecting the district office to the lower levels and improving the planning and management of sub‐district health services.^27^ Within the health sub‐district there are health centres (II, III and IV) and village health teams.

This study focuses on the district, which is the highest local government level. The District Council is the main governing body of the district. Meanwhile, there are two power centres. There is the political wing comprising of council members, under the Local Council‐5 (LC‐5) chairperson (comprising of council members), which approves policies and budgets and is responsible for standardization and enforcement of policies in the district following public standing orders and the Local Government Act. The council members form different committees, the District Health Committee (DHC) being one of them which is the political governing body of the health sector. The technical wing is led by the Chief Administrative Officer (CAO) and chairs the Technical Planning Committee (TPC) which determines the district priorities. The District Health Team (DHT) consisting of the heads of district health programmes assists the District Health Officer (DHO) in implementing the health policies and plans and is supported by the DHMT. The DHMT is a larger body that includes health facility managers, members from other departments, political and administrative district managers and representatives from private healthcare providers and local NGOs (Figure 1, Table 1).^19^, ^28^

**TABLE 1:** Key decision‐makers in the district^19^, ^28^

Actor	Role
District Council	is the District Local Government Council that governs the district and includes different district committees
Local Council Chairperson‐5 (LC‐5)	is the elected chairman and the head of the legislative or political body who approves policies and budgets that are responsible for standardization and enforcement of policies in the district following public standing orders and Local Government Act
Chief Administrative Officer (CAO)	is the head of the administrative body, he has wide influence and presides all departments in the district.
Technical Planning Committee (TPC)	is a technical committee headed by the CAO and is responsible for planning and determining the district priorities
District Health Committee (DHC)	one of the district committees that acts as the political governing body of the health sector
District Health Team (DHT)	the executive team who implements the health policies and health sector plans and consists of the heads of district health programs
District Health Manager Team (DHMT)	is a larger body, which includes the DHT, health centre managers, other departments' members, political and administrative district managers, representatives of the private health sector and local NGOs
District Health Officer (DHO)	is the head of the health department who manages both the DHT and DHMT

According to the Local Government Act 1997, the decentralisation policy in Uganda states that the powers and responsibilities of the central government for public services planning and delivery should be transferred to the local governments.^29^, ^30^, ^31^ The Ugandan experience was successful in comparison to other LMICs that used decentralisation as a health sector reform policy, especially in the scale and scope of the decision‐making powers devolved to the local level.^28^, ^29^, ^30^, ^31^, ^32^ Yet, the DHMTs in Uganda still argue that more power and decision space are needed to develop innovative ways of improving health services.^28^ When studying decision space, it is not only important to consider the policies and regulations that mandate authority but also to explore the processes that influence the use of decision space and to investigate the relationship dynamic between them.^3^, ^32^, ^33^ Although there is a growing body of literature showing how decentralisation can lead to the transfer of decision‐making power and discretion for HRM functions from central governments to local levels, the impact of decentralisation on HRM has not yet received adequate research attention.^14^ Research on decentralisation has also focused on who has more control over decision‐making but not on how this control is used.^34^, ^35^ Therefore, the aim of this study is to examine the use of the DHMTs' reported decision space related to HRM functions in Uganda. It builds upon Alonso‐Garbayo's work of 2017 which assessed the decision space that managers have in six HRM functions and compared the roles allocated by Uganda's policy and regulatory frameworks with the actual room for decision making that the DHMTs were perceived to have.^28^ This paper looks at the decision space reported by the DHMTs in Uganda, how they use it and identifies areas for improvement. It also explores factors supporting or limiting the DHMTs' use of their decision space and how these might influence performance and service delivery in districts.

## 3 METHODS

A mixed‐methods approach was used to examine the DHMTs' reported decision space for HR‐related functions in three districts in the Rwenzori region of west Uganda: Bunyangabu, Ntoroko and Kabarole. These three districts were part of the PERFORM2Scale programme (2017–2021) which is scaling‐up a district level management strengthening intervention in Ghana, Uganda and Malawi. It supports groups of DHMTs in each country through a series of action research cycles to identify workforce‐related problems and develop integrated strategies that can be included in the annual district plans using their available resources. (See https://www.perform2scale.org/) Kabarole district was chosen since it was among the participating districts in the pilot PERFORM study (2011–2015). However, in 2016, Kabarole district was reorganised, creating Bunangabu district and a smaller Kabarole district. Therefore, Bunyangabu was also selected to be part of the study. Ntoroko district was selected because of its geographic proximity to Kabarole and Bunyangabu to facilitate inter‐district meetings. The three districts were also recommended by the programme's National Scale‐up Steering Group (NSSG) in Uganda to facilitate inter‐district learning. The following section provides a brief overview of each district, guided by the situational analysis done by the PERFORM2Scale research team for this district group.

Bunyangabu district was carved out of Kabarole district in 2016 and has been effectively operating since July 2017. The extended DHMT is comprised of 20 members including core DHT members, and health facility in‐charges, among others. The team includes three women and 17 males. The DHMT identified the following HRM problems in the district: understaffing of technical health workers at health facilities; inadequate/incompetent leadership at lower‐level health facilities; high absenteeism rate among staff; inadequate capacity for Health Unit Management Committees to effectively monitor and evaluate health unit activities; and inadequate staff performance management in all health facilities.

Ntoroko was carved out of Bundibugyo district and granted district status by the act of parliament in 2010. The extended DHMT has 24 members including core DHT, focal persons of various service delivery areas and health facility in‐charges. There are three women and 21 men in the DHMT. The district has 123 posts of which 88 (71.5%) are filled. HRM problems as identified by the DHMT include leadership and management gap at the district and health facility levels; poor planning and implementation of core functions within the DHT/DHMT, that is, data review meetings, data collection, support supervision visits; knowledge gap in recording and compiling immunization data; six of the eight DHT members are in Acting position; and challenges in conducting DHMT meetings.

In Kabarole district, the extended DHMT includes 22 members: five core DHT members, four hospital in‐charges, two HSD In‐charges and 11 program heads/Focal persons and partners. The majority of DHMT members are males (15 out of 22). The DHMT identified some HRM problems, these include irregular weekly, monthly, and quarterly DHT/DHMT meetings; inadequate support supervision conducted by DHT to health facilities; lack of iHRIS system in the district Health department; poor performance management at the district and health facility level; and limited capacity of health workers to analyse and use data for planning purposes.

The study used self‐assessment questionnaires and focus group discussions (FGDs); an approach that has been used in other studies.^28^ In each district, three or four DHMT members (district health officers, maternal and child health officers and biostatisticians) and the HR officer from the Local Council were purposively selected to participate in the survey and FGD. All have several years of working in the district, are knowledgeable about HR and service delivery in the district and are in decision making positions. There were four male participants in Bunyangabu; three males and one female in Kabarole; and only three males in Ntoroko. The HR officers from the three districts were all male. Initially, each district group was asked to fill in a self‐assessment questionnaire, and each group reached a consensus score regarding their level of decision space for each HRM function as they perceived it. ‘Full’ refers to the DHMTs having complete control over the function, ‘some’ indicates partial control, and ‘none’ is having no control at all. These scores are presented in Table 2. These responses were then discussed in detail in each FGD. The participants were asked to reflect on their reported decision space and its use based on their recent experience before the implementation of the PERFORM2Scale intervention in 2017. Participants were asked to provide justifications for their choices, to describe the process of each function and how they use their decision space, the consequences of the decision space they have, and how that could influence their performance. Finally, participants identified factors that support and hinder their decision space and reflected on their performance and the level of authority they need in order to promote workforce performance, strengthen management and improve service delivery. To note, the FGDs did not modify the score on Table 2, and the final assessment was purely the perception of the respondents without the researchers' inputs. All discussions took place in the districts, they were conducted in English, recorded after consent from all participants, and lasted for approximately 1.5 h. The recorded FGDs were then transcribed, and each transcription was imported to NVivo qualitative data software for analysis.

**TABLE 2:** Decision space of HRM functions in three districts in Uganda


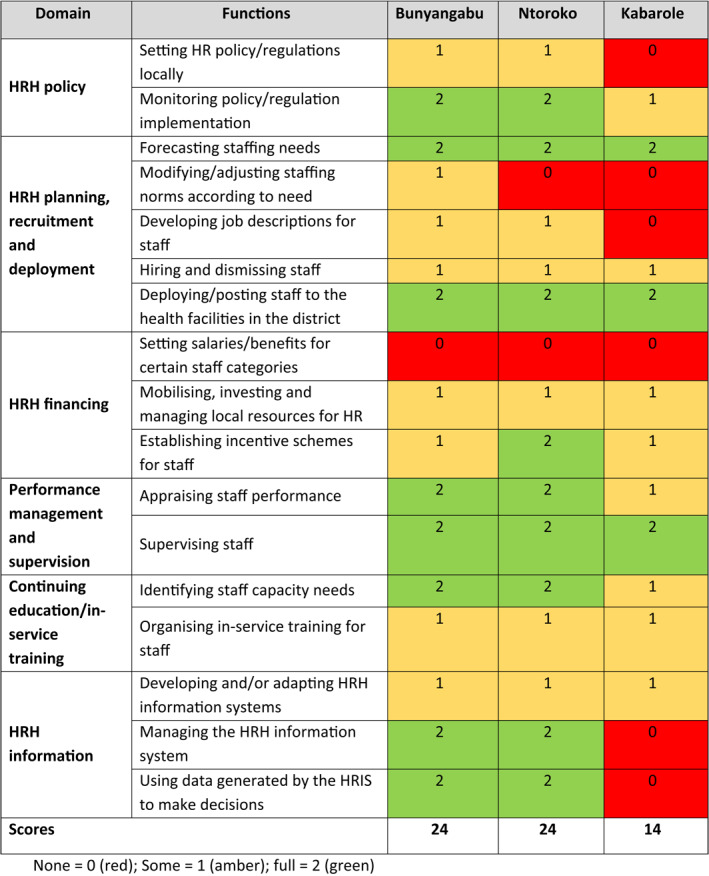

**FIGURE 2:**
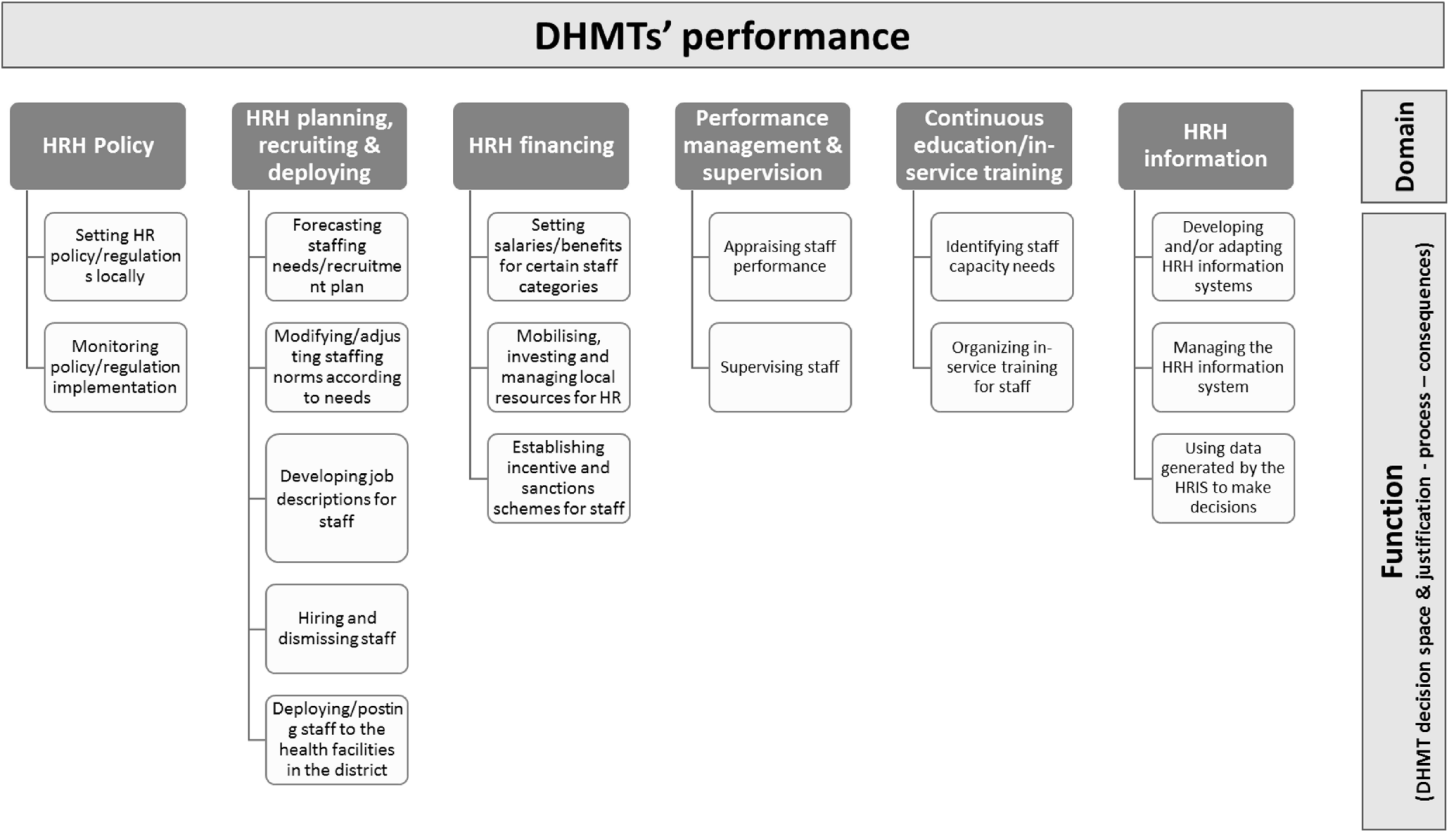
Framework for analysis

Analysis of the FGDs was guided by a framework developed by the research team (Figure 2) and deductively extracted from the topic guides used in the FGDs (Appendix A in supporting information S1). The framework is formed of six domains: HRH policy; HRH planning, recruiting and deploying; HRH financing; performance management and supervision; continuing education/in‐service training; and HRH information. These domains were adapted from a tool used to assess the decentralisation of health services in Africa^36^ and used by Alonso‐Garbayo and colleagues to assess the DHMTs' decision space in Uganda.^28^ Each domain includes a number of HRM functions that were specifically used to explore the DHMTs' decision space and the level of control they have over each function (Figure 2).

NVivo qualitative data analysis software was used to conduct a thematic analysis for each district. Within each domain/high level code, functions/themes were identified and text relevant to any of these functions was highlighted and coded. This was used to generate insights about the level of control that the DHMTs have over HRM functions and how they use it, and finally to compare the findings across the three districts.

## 4 ETHICAL ISSUES/STATEMENT

Ethical approval for this research was obtained from the Ethics Committees at both Liverpool School of Tropical Medicine (Ref. 17‐046) and Makerere University School of Public Health (Ref. HDREC539). All participants signed a written consent form before the FGDs after having been thoroughly informed about the research. Data were managed, stored, analysed and presented ensuring full confidentiality.

## 5 RESULTS

The responses from the self‐assessment questionnaire are shown in Table 2. The three DHMTs reported varying levels of control over HRM functions, ranging from ‘none’ to ‘full’. The DHMTs in Bunyangabu and Ntoroko reported a wider decision space than their peers in Kabarole. The latter reported a limited level of control over setting HR policy locally, developing job descriptions and all functions of the HRH information domain.

The three DHMTs reported ‘full’ control over the performance management and supervision domain and its functions. They reported ‘full’ control over another four HRM functions: monitoring policy implementation, forecasting staffing needs, deploying staff to health facilities, and identifying staff capacity needs. They reported ‘some’ control over other functions, for example, developing job descriptions, mobilising resources for HR and organising in‐service training.

All respondents reported limited authority for three functions: modifying staffing norms, setting salaries for certain staff categories, and developing a HR information system (HRIS). However, they tried to overcome these limitations by adjusting some HR policies and tools locally, better utilising available resources and adapting the HRIS to their local context. These, in turn, improved workforce performance and facilitated service delivery and outcomes in health facilities, as reported by the DHMTs during FGDs.

The next section presents the findings from the FGDs which explored the survey responses in detail. It is structured around the domains and functions in the analytical framework. For each function area, a brief overview of the reported DHMT's decision space and how it is used to make and implement decisions is provided. Factors supporting and limiting DHMTs' decision space are also considered in each function area.

## 6 HRH POLICY

### 6.1 Setting HR policy/regulations locally and monitoring policy implementation

Policies are developed at the central level and the DHMTs do not have the power to develop an entire policy. However, Bunyangabu and Ntoroko DHMTs reported having ‘some’ authority to adjust HR policies to their local needs and context which makes the policies easier to use and for the staff to comply with, and which helps improve service delivery in the health facilities. In Kabarole district, the DHMT claimed to have no control over this function.

In Bunyangabu, the DHMTs argued that the process of adapting a local policy could take one to three months. First, it is proposed at DHT level before the DHT takes to their Committee for Health which discusses it and then submits to the District Local Council for approval, so it can be implemented to address local problems in the district.

…the HR at the upper level [national] was weak in the rewards and sanctions. We sat down and decided to develop our own rewards and sanctions at district level, I mean at departmental level which of course ideally would have been at the Chief Administrative Office (CAO)'s level. So, we passed it for blessing […] So we were able to have our functional rewards and sanctions at HCIV, and at departmental level at DHOs office, which was fully authorized (Bunyangabu).

Once the policies have been developed and approved at the central level, it is the core mandate of the DHMTs [at district level] to ensure that there is compliance by staff to these policies. The DHMTs in Bunyangabu and Ntoroko reported ‘full’ authority in monitoring the implementation of policies and regulations. However, in Kabarole they reported only having ‘some’ control and cannot always complete the implementation process due to bureaucracy.

… some policies are beyond us, we check the process, but we cannot complete it due to bureaucracies … Like for sanctioning […] we usually get complaints from the lower facilities then come to the health sub district, then to the DHT, and then the DHT forwards it to the CAO. So that where we stop […] we stop at forwarding (Kabarole).

## 7 HRH PLANNING, RECRUITMENT AND DEPLOYMENT

### 7.1 Forecasting staffing needs/recruitment planning

All three DHMTs reported having a ‘full’ mandate to forecast staffing needs. They can identify staffing needs for each department within a certain period and forecast them, with a view to filling staffing gaps that hinder service delivery at health units.

**FIGURE 3:**
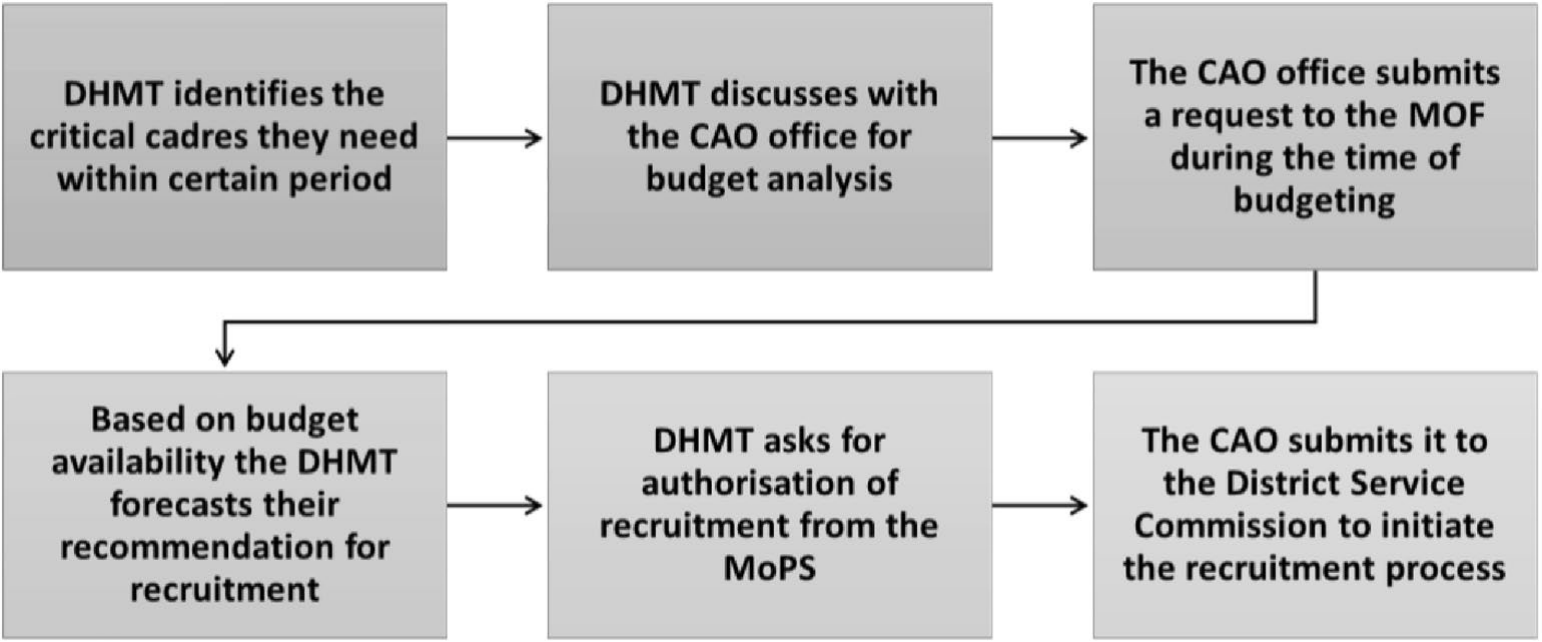
Recruitment plan process

The DHMTs described developing a district recruitment plan every other year (Figure 3). It is mandated to identify the critical cadres that will be needed within a particular period and to recommend recruitment, according to the staffing norms (e.g., in Ntoroko there should be three nurses and two midwives in HCIII). The DHMT first discusses the available staffing budget with the HR department at the CAO's office, and then decides who can be recruited. The HR office then submits a request to the Ministry of Finance at the time annual budgets are set, and accordingly, the DHMT can forecast their recommendation for recruitment and ask for authorisation for recruitment from the Ministry of Public Service (MoPS). After gaining the authorisation from the MoPS, the CAO's office submits the recruitment request to the District Service Commission (DSC) which in turn initiates the recruitment process and advertises positions (Figure 3).

### 7.2 Modifying/adjusting staffing norms according to needs

It is the role of the MoPS to set the staffing norms in collaboration with the Ministry of Health (MoH). So, the DHMTs in Ntoroko and Kabarole reported having ‘no’ control over changing the norms. However, once the staffing norms are approved for a particular level of facility, the DHMTs in the district may have some authority to customise it. The DHMT in Bunyangabu reported having ‘some’ control over this function of adjusting the staffing norms through transfer/secondment to overcome the shortage of the staff.

…the staffing norm for HCIII for example is … there are supposed to be two midwives, but you may go to a facility which delivers extremely a higher number and it overburdens the midwives. So, at the district level we can identify, and we can pick one midwife from somewhere where there is less work to another. That's why we said at least we have some control here (Bunyangabu).

### 7.3 Developing job descriptions for staff

Formal job descriptions are developed at the central level by the MoPS, and DHMTs have no control over their development. However, the DHMTs in Bunyangabu and Ntoroko exercise some flexibility when they recruit new staff, they add more duties to the job description depending on their department's needs. The DHMTs perceive schedules of duties as being synonymous with job descriptions. Most staff are happy to take on the additional duties to maintain their skill levels or because of the available incentives, for example, the DHMT in Ntoroko reported attending workshops in return for taking on the role of malaria focal person.

That kind of flexibility for a manager to be able to give an officer extra duties has facilitated service delivery in cases where for example the district does not have wage to recruit the person who would actually be performing such kind of a role … (Ntoroko).

### 7.4 Hiring, deploying and dismissing staff

The DHMTs in the three districts have ‘some’ decision space with regard to hiring staff. They reported this is adequate since they can initiate the recruitment process by identifying staffing needs and making recommendations, but they do not complete the process; once they make their recommendations, the CAO takes the request forward to the DSC (Figure 3).

The hiring process starts at the DSC, with the DHMTs participating when the DSC advertises positions. The DHMTs assist in identifying the necessary characteristics and qualifications for the role, devising interview questions and interviewing applicants.

…if it is a position to be filled in the health department, it is a technical officer who is selected from the health department to be in the interview… And where we do not have technical officer within the department we outsource, we go to the regional referral hospital or Ministry of Health or we can even borrow from another district (Kabarole).

As shown in the recruitment plan process (Figure 3), hiring depends on budget availability; if it is not available the DHMTs cannot hire. Given the intermittent government recruitment freezes, DHMTs also need to request permission from the MoPS to recruit more staff.^37^

The DHMTs in the three districts have ‘full’ authority for deploying staff to health facilities within their district. When people are newly recruited, the DHMTs send their recommendations to the CAO on where these people should be deployed. They can also make recommendations for transfers and redeployment of any existing staff according to these needs. In most cases, the DHMTs' recommendations are fully acted upon by the CAO.

Staff dismissals are initiated by DHMTs. However, the authority to dismiss an employee lies at a higher level. It is a lengthy and bureaucratic process as described by the DHMT in Notoroko, and politics often play a role in it since some staff members are 'untouchable’. When a staff member is not performing to expectations, the DHMTs will issue a warning. If the staff member continues to underperform, the problem is forwarded to the CAO who submits it to the DSC for further action, and possibly dismissal, as reported by the DHMT in Bunyangabu district.

Although most DHMT members feel they have adequate levels of authority with regard to hiring and dismissing staff, the DHMT in Ntoroko argued for more control over both processes.

…if this was a private entity, like how the private health facilities work and how they have the full authority to recruit and also to dismiss, if it was like that it would somehow improve service delivery. Because […] these processes are too long and somewhere you can't stretch further and to ensure that the person is really out of the system [dismissed]” (Ntoroko).

## 8 HRH FINANCING

### 8.1 Setting salaries/benefits for certain staff categories

The DHMTs have no control over setting salaries and benefits for any staff categories. Salaries are set by the MoPS, and the HR department is responsible for implementation in each district. The policy states that for anyone to qualify for a salary he/she must work for at least 15 days per month. Therefore, the DHMTs have a supervisory role in ensuring that every facility manager submits their monthly return of staff attendance. DHMTs can then decide who is to be paid that month and forward their names to HR and the DHO's office will execute without reference to the CAO.

### 8.2 Mobilising, investing and managing local resources for HR

The DHMTs in the three districts have ‘some’ authority over mobilising and managing local resources for HR. It is not entirely their remit; resource mobilisation must be endorsed by the CAO and it is the DHMT's full mandate to initiate conversations with the government or implementing partners (IPs) on the issue. Some resources are directly managed by IPs, while others are managed through the district finance management system and the Department of Health. Other resources come directly to the district to fund different activities such as mentorship and training.

The DHMTs reported looking for opportunities for funding activities within the district. They then develop plans for activities and submit these to the CAO to negotiate with the IPs who are working in their district. The Ntoroko DHMT explains how this approach has helped support nutritional assessment in their district:

…through appraisal system, when we realize some [skills] gaps in our health workers, we write a concept to the developing partners for example we have UNICEF, you find a gap in nutritional assessment, people don't know how to do nutritional assessment, so you take the concept to UNICEF they support us, we do mentorship and training (Ntoroko).

### 8.3 Establishing incentives and sanctions schemes for staff

The DHMT in Ntoroko reported having ‘full’ control over establishing incentives schemes for staff, whereas, in Bunyangabu and Kabarole they have only ‘some’ level of control. However, the three districts stated ‘full’ control over the implementation of these incentives and sanctions schemes.

The DHMTs operate under a rewards and sanctions framework, established by the MoPS. It mandates some DHMT members to sit on the Rewards and Sanctions Committee so they can identify rewards for their well‐performing staff. These rewards might be in the form of transportation and training opportunities or workshops, depending on the availability of resources. Rewards can also come in the form of recognition including through assignments. For example, if a nurse is recognised as being active, he/she can become the focal person for the malaria or HIV programme. The only form of sanction mentioned was dismissal which has been described above.

## 9 PERFORMANCE MANAGEMENT AND SUPERVISION

### 9.1 Appraising staff performance

In Bunyangabu and Ntoroko, the DHMTs have ‘full’ control of staff performance appraisal, whereas Kabarole DHMT reported they only have ‘some’ control.

The DHMTs in Ntoroko and Bunyangabu reported that each health department is required to appraise its staff at the end of each year. The DHO sets performance plans then the facility manager, in consultation with other staff, set staff targets. The DHO appraises both the DHMT members and those at the HCIV. The facility managers appraise the health workers in their facilities. The DHO is appraised by the CAO, as reported by the DHMT in Kabarole.

Furthermore, the DHMTs monitor performance regularly through their visits to the facilities, attending meetings with the facility managers, monitoring staff progression and identifying any challenges. At the end of the year, the DHMTs complete the performance reports, which they submit to the CAO. Rewards and sanctions are determined accordingly. The CAO also supports the DHMT's decisions on performance‐based rewards and sanctions and s/he also helps with capacity building through mobilising resources as reported by Bunyangabu district. Monthly health centre reports, generated by the health information system, also help with the performance appraisal (see HRH information).

### 9.2 Supervising staff

Staff supervision is within the core mandate of the DHMTs, and the three districts reported ‘full’ control. This is done through integrated supportive supervision visits that help identify capacity gaps and ways to address them such as mentoring. The HR officers play a vital role in creating awareness of HR issues—such as staff development—among DHMTs and work closely with them to build capacities.

The DHMTs provide support, on‐site training and mentoring to staff in the health facilities to help improve performance. For example:

…there was a time we were reporting a high number of TB cases in […] but it was because the lab person was having an issue with reading the slides. […] the TB focal person went there; he realized the anomaly in the number of TB microscopic cases. On trying to read the slides that had been read by the lab assistant [her] realized that they were actually not positive, but it was the lab assistant […] It was like he was reading false positives […] So the decision we had an on‐site mentorship on how to read the TB slides and from then the number of TB microscopic from Kabarole actually dropped (Kabarole).

## 10 CONTINUOUS EDUCATION/IN‐SERVICE TRAINING

### 10.1 Identifying staff capacity needs

The DHMTs in Bunyangabu and Ntoroko have ‘full’ control over identifying their staff capacity needs through appraisal and supervision process. During a meeting held between the supervisor and the staff member, any issues or capacity needs relating to skills and performance are discussed. For example, there was a laboratory assistant in Bunyangabu district who struggled with using a specific machine:

We have had the distribution of CD4 [a test provided for people with HIV] machines and yet we have laboratory assistant who we know they are not so much competent in use of those machines [...] So there since you have decided to put this one in their job schedule you have to make sure that you identify that one as a need, train them […] in order for them to catch up with the need (Bunyangabu).

A plan for support is designed and the staff might be attached to a larger facility to receive on‐site training and address their performance gaps. However, the DHMTs still do little to build capacities because of the shortfall in the government budget. The DHMT in Kabarole recognised the need to have more control over this function.

### 10.2 Organising in‐service training for staff

The DHMTs in the three districts have ‘some’ level of authority over organising in‐service training for staff, depending on the nature of the training needed. If the required skill is available at the facility, then the staff member can be mentored on‐site. If not, then this will require the allocation of scarce resources and time away from the facility for further training which DHMTs do not always have control over.

The DHMTs use the available resources in the district (either the annual district budget or the Primary Health Care (PHC) funds) to train their staff, especially if there is no partner to support training. They identify key people who are skilled in an activity, and a temporary mentoring placement to a different facility takes place and when they come back, they can train other staff in the facility.

…if someone has been identified to go for a workshop on TB, when they go back to the facility, they are supposed to organize Continuous Medical Education (CME) so that they pass on this knowledge to their colleagues they left at the facilities. So ideally each facility should have a CME schedule where they can share knowledge they have got from other places (Bunyangabu).

However, this might need the authority to move a person to two or three facilities or to enable them to visit the facilities, which the DHMTs may not have.

## 11 HRH INFORMATION

### 11.1 Developing and/or adapting HRH information systems

The DHMTs reported having no control over developing an HR information system (HRIS). However, they have ‘some’ authority to adapt the national MoH HRIS as reported by the three districts. They adapt it to their local needs and use the available indicators to report to the MoH.

We have some decision space particularly for adapting because for the purposes of reporting and handling things in line with our line ministry we may not be able to develop a parallel system. We rely on the national system which has been developed, but as a district we can adapt that so we have the capacity to adapt the HRM information system from ministry of health and usually they give us full rights to use the system and report to them. But developing, no (Bunyangabu).

The HRIS is functional in the three districts. It is an online system in the DHO's office, and both the HR office and the DHMTs have access to it. The monthly attendance returns, transfers, and annual leave requests are fully updated on the system.

### 11.2 Managing the HRH information system and using data generated to make decisions

In Bunyangabu and Ntoroko, the teams have ‘full’ authority over managing the information system, as well as using its data for evidence‐based HR decision making. The HR officer can analyse the data in the HR information system to identify the current staffing gaps in each facility, forecast staffing needs, and develop an HR plan.

The DHMTs used to regularly visit the facility to monitor absenteeism, but now can easily look at attendance to duty using the HRIS. Every month the facility manager sends an attendance summary which includes authorised and unauthorised absences to the DHMTs. The DHMTs enter this data into the HR system.

…you get very high absenteeism rate sometimes but if you go on ground to check you find some of these people were allowed to be off or officially maybe for training purposes but because that was not being captured, you find a very alarming absenteeism rate. So we went on engaging the in‐charges [facility managers] […] we had several meetings on how to fill his form, what it means to put those indicators […] But gradually we are seeing it improving in the quality of data which is submitted […] generally it is improving the service delivery in those facilities (Bunyangabu).

In Kabarole, the DHMT do not use the system to generate data for decision making because of staffing issues, as explained below. However, they plan to reactivate the system and use it to manage HRM functions.

the system came in play when Bunyangabu and Kabarole were still one district. So, the person who was running the system in [District B] when there was a division of the district, the person was moved to Bunyangabu. That was actually the biggest problem. So, the other people who were left behind who were supposed to run the system did not fully take it on (Kabarole).

## 12 DISCUSSION

Analysing decision space has frequently been used to examine the level of control delegated to local levels of the health system in decentralised contexts.^7^, ^9^, ^11^, ^38^ The main assumption is that the more decision space there is available, the more innovative, adaptive, responsive and efficient the decisions made by local governments are, which would strengthen performance management and ultimately improve service delivery.^8^, ^9^, ^39^

In this study, analysing decision space as reported by the DHMTs, provided a real‐world view of the degree of decentralisation regarding HRM functions in the health sector at district level in Uganda. It explored the reported decision‐making powers transferred from the central to local government and how DHMTs use this power to improve workforce performance. It is a baseline study to report the DHMT's decision space early with the start of PERFORM2Scale intervention. The planned future research will re‐examine it again with the last cycle of the intervention in the same district group to explore if the intervention increased decision space for the DHMTs.

The decision space available for the DHMTs in the three studied districts varied between ‘none’ and ‘full’ control over some HRM functions. All DHMTs reported full control over the performance management and supervision domain and its functions; monitoring policy implementation; forecasting staffing needs; deploying staff to health facilities and identifying staff capacity needs. However, the decision space for all DHMTs was narrow for developing job descriptions; mobilising resources for HR; and organising in‐service training. They have no control over some HR functions that are set at the central level such as modifying staffing norms, setting salaries for certain staff categories and developing an HRIS. Noticeably, the DHMTs in Bunyangabu and Ntoroko reported that they could exert more control than their peers in Kabarole. The DHMT in Kabarole reported having no control over setting HR policy locally, developing job descriptions, and all functions of the HRH information domain. Kabarole district was restructured in 2016 and was smaller with a change in district management staff from the Kabarole district that participated in the PERFORM project. Any effects of the PERFORM2Scale management strengthening project on widening DHMT decision space is therefore diluted. Nevertheless, all DHMTs tried to overcome their limited decision by adjusting some HR policies and tools locally, better utilising available resources and adapting the HRIS to their local context, which in turn, improved workforce performance at the district level.

The real benefit of decentralisation is dependent on allowing the DHMTs to use the decision space provided to them in applying required changes at local levels.^38^ In Uganda, devolving authority to DHMTs allows them to use the decision space available to them to overcome the limited authority they experience over some HRM functions, and to adopt flexible approaches and be responsive to their local needs, which in turn, helps improve the quality of care.^1^, ^13^ The DHMTs in Uganda organised transfers and relocations for certain roles to cover their staffing gaps and this helped them in deploying some staff and using them for other staff development. Transfers and placements were their strategies for bridging the skills gap among their staff. They allocated qualified people to support health facilities through providing in‐service mentorship and training. Expanding schedules of duties was another approach used by the DHMTs to overcome the limited control they have over ‘developing job descriptions’ which helped them cover extra activities needed in the district. Being able to customise policies and tools to their local needs made it easy for them to follow and implement those policies and bestowed a sense of ownership. This flexibility for the DHMTs helps them prioritise their local needs and overcome the bureaucracy in the system and lengthy recruitment processes. It helps them maintain adequate performance when the district does not have the budget to recruit new staff, to promote efficiency and effectiveness, and to improve service delivery.

The DHMTs' decision space can be influenced by the resources available in the district, their management competencies, and the overall local context.^34^ In fact, the lack of resources remains the most significant factor. The literature suggests that the DHMTs need to be granted not only the power but also the resources to be able to manage their health workforce.^34^, ^35^, ^36^, ^37^, ^38^, ^39^, ^40^ It has been reported that the central government in Uganda did not provide districts with the funds needed to accomplish the activities transferred to them as part of decentralisation, and the districts cannot generate these funds locally. Unsurprisingly, in this study, the DHMTs reported having minimal decision space for ‘HRH financing’. They have no authority to set salaries or benefits for any staff categories. They only have ‘some’ control over establishing incentive schemes which are mostly non‐monetary due to scarce financial resources. They can also mobilise and manage local resources which is frequently facilitated by IPs, and they often rely on IPs to fund incentives as well. DHMTs in Uganda, by recognising and using the decision space available to them, can make better use of the scarce resources available to them, and ultimately better manage their workforce despite the limited resources available.

Lack of funds has a negative impact on HRM functions. Despite the DHMT being able to forecast staffing needs, recruitment may often be limited due to lack of funds. It also limits the DHMTs' authority to provide training and build capacities among their staff.^28^ Ideally, they should provide a quarterly training plan, but with limited resources they can only provide training once or twice a year. The DHMTs also reported that the districts suffer from insufficient skills and capacity of some staff in health facilities, for example, the reported cases of the lack of nutritional assessment and false positive testing of TB cases by some staff, but the DHMTs tried to overcome this by conducting on‐site mentorship to build capacities among staff. This goes in accordance with recommendations from the literature that the available decision space to managers is better used when it is accompanied by capacity building and better accountability.^9^, ^17^, ^18^

Politics and bureaucracy also play a role in limiting the decision‐making powers transferred to the DHMTs.^3^ On one hand, politics hinder the implementation of some policies for example, sanctioning, especially where some staff members are ‘untouchable’ as reported by one DHMT. On the other hand, governmental bureaucracy resulted in inflexibility and delay in the implementation of some functions such as recruitment, dismissal and sanctioning, the DHMTs often submit their plans, but actions take time to be implemented. In order to be more adaptive and responsive to their local health system needs, DHMTs need a wider decision space and strengthened capacity to use this decision space. Clarifying the role of the central government and the relationship dynamics with the local level would also help eliminate political interference and support the DHMT to better use their available decision space.^18^

## 13 RECOMMENDATIONS FOR POLICY AND PRACTICE

Increased decision space is not an end in itself, but rather a management approach to improve workforce performance and promote service delivery. To institutionalise decentralisation, there is a need to grant more decision‐making power to the local level, to build the financial and HR capacities and strengthen the management competencies for the effective use of decision space and wise allocation of available resources. For successful implementation of decentralisation, policies need to consider not only ‘de jure’ decision space but also provide implementation plans that support ‘de facto’ functioning, where the DHMTs can better use their available decision space.^7^, ^11^ Effective decentralisation also needs a clear set of roles and responsibilities for all levels in the decentralised health system to determine the real degree of decision space available to the local level and alleviate bureaucracy in the health system.

## 14 LIMITATIONS OF THE STUDY

There are some limitations to this study that need to be considered. It was not possible to get detailed explanations for all of the responses in the self‐assessment questionnaire during the FGDs due to time constraints for example, the justifications behind some reported levels of decision space for the DHMTs, and information regarding the tension between the technical and political wings. In particular, data on decision space was based on the perceptions on the DHMT members, and neither the local nor central government officials, who might have different perceptions, were included in the study. Although, the FGDs were conducted with three or four DHMT members, they were purposively selected based on their knowledge and experience of HR and decision making within their district. They reported on their routine work, and they were knowledgeable enough to be able to provide the required information. By including the HR officer, we were able to get valuable insights about HR functions in each district. Finally, this study did not examine the performance outcomes because it is a baseline study; however, future research will look at these outcomes with the second round and the collection of the end‐line data for the same district group of PERFORM2Scale.

## 15 CONCLUSION

Decentralisation provides a critical opportunity to strengthen HRM in LMICs. In Uganda, devolving authority to the local government helped the DHMTs be more responsive to their local needs. Analysing decision space for HRM functions helped identify areas where the DHMTs can change or improve their actions. Despite the existence of policies and regulations, a lack of resources, bureaucracy, local politics, and skills gaps remain major challenges to the use of available decision‐space by the DHMTs. Arguably, in a continuation of the process of decentralisation, the DHMTs in Uganda should be given more discretion, or increased decision space, to manage their staff in a way that is appropriate to local needs. However, such action should be complemented by further development of management competencies to enable them to become more resourceful and responsive to these local needs.

## CONFLICT OF INTEREST

The authors report grants from European Union's Horizon 2020, during the conduct of the study.

## ETHICS STATEMENT

Ethical approval for this research was obtained from the Ethics Committees at both Liverpool School of Tropical Medicine (Ref. 17‐046) and Makerere University School of Public Health (Ref. HDREC539).

## Supporting information

Supporting Information S1

## Data Availability

The data that support the findings of this study are available on request from the corresponding author. The data are not publicly available due to privacy or ethical restrictions.
