# Supplementary material for: Management of human resources for health in health districts in Uganda: A decision space analysis
Source: Int J Health Plann Manage. 2021 Oct 25;37(2):770–89. doi: 10.1002/hpm.3359 (PMC9298089; doi:10.1002/hpm.3359)
Supplement: Supplementary file 1 — Supporting Information S1 [file HPM-37-770-s001.pdf]

GUIDANCE TO USE THE TOOL TO ASSESS DHMT DECISION-SPACE FOR HUMAN RESOURCES MANAGEMENT

To understand the DHMT's decision-space for human resource management (HRM) a self-assessment by DHMT members is conducted. Two dimensions of the decision space are assessed: **perceived authority for HRM** and **reported actual practice in HRM**.

The assessment will be carried out in each district of the DG1 before starting implementation of the first cycle of MSI in Y2 and at the end of the last cycle of MSI in Y4.
The tool contains two tables:

- Table 1: Perceived authority
- Table 2: Actual practice

The objectives of using this method in the PERFORM2Scale (P2S) project are:

- In Y2:
    - To define a baseline against which to measure changes in the perceived decision space and its use following the P2S intervention
    - To help us get a deeper understanding of the DHMT functions with regard to human resource management
    - For the DHMT to identify areas of human resource management where they have the authority to act and to reflect on their actions in these areas
    - A potential outcome of this exercise is that DHMT may choose to improve their actions in areas where change or improvement is required
- In Y4:
    - To be able to measure changes in the DHMT perceptions and use of HRM decision space following the P2S intervention

The CRT will need to first explain to the DHMT the purpose of undergoing the self-assessment exercise as well as its potential benefits for the team and agree with them a convenient time and place when the self-assessment will be done.

DHMTs will be requested to select three or four members to participate (one should be the officer who is most involved in HRM). The same four members should participate in both elements of the tool. Also, where possible, same members should participate in the assessments in Y2 and Y4.

How to use the tool for Self-Assessment of perceived decision space of DHMT in Human Resource Management

1. Print Table 1 for the DHMT
2. Enter the name of the district, name and position within the DHMT of each participant, your name/s, date and the reference number on the first sheet of the tool
3. Ask the DHMT to discuss and reach consensus about answers for perceived authority table and put a "X" in the appropriate column (i.e. none, some or full); CRT members should facilitate the discussion ensuring there are no dominant voices and supporting full participation from all participants but avoid leading or influencing participants' decisions.
4. Once completed, ask the participants to provide an explanation of why they have chosen that option writing it down in the 'comments' box. Make sure that they give explanation for all functions

How to use the tool for Assessment of DHMT actual practice in Human Resource management

This element consists of a focus group discussion with the same participants who completed the previous one. The discussion will be recorded after obtaining consent from each of the participants. The form will act as an interview guide and a note-taking tool for the interviewer.

The interviewer should base the questions on the perceived level of authority for each function reported before: i.e. "based on the "some" or "full" level of authority you said you have to …… (specific function) …" (NOTE: if their answer was "none" skip that function and move to the next one)

- What is DHMT's role in conducting this function?
- What is the result of performing this function?
- What is your assessment/reflection on the DHMT's role in performing this function?
- Please use the 'comment' column to enter explanatory notes about their answers.

Once completed thank all participants for their time.

Name of district:	
Completed by (all names):	1. Name…………………………………………… Age………. Gender …….. Post……………………………………………………………….<br>2. Name…………………………………………… Age………. Gender …….. Post……………………………………………………………….<br>3. Name…………………………………………… Age………. Gender …….. Post……………………………………………………………….<br>4. Name…………………………………………… Age………. Gender …….. Post……………………………………………………………….
Interviewer(s):	1. Name…………………………………………….<br>2. Name…………………………………………….
Date:	
Reference Number:	

Table 1. Self-assessment of DHMT perceived authority in Human Resource management

1. Domain	2. Function	3. Level of authority (mark with an X)			4. Explanation of choice
		None	Some	Full	
**HRH policy**	Setting HR policy/regulations locally				
	Monitoring policy/regulation implementation				
**HRH planning, recruiting and deploying**	Forecasting staffing needs				
	Modifying/adjusting staffing norms according to need				

	Developing job descriptions for staff				
	Hiring and dismissing staff				
	Deploying/posting staff to the health facilities in the district				
**HRH financing**	Setting salaries/benefits for certain staff categories				
	Mobilising, investing and managing local resources for HR				

	Establishing incentive schemes for staff				
**Performance management and supervision**	Appraising staff's performance				
	Supervising staff				
**Continuing education/in-service training**	Identifying staff capacity needs				

	Organizing in-service training for staff				
**HRH information**	Developing and/or adapting HRH information systems				
	Managing the HRH information system				
	Using data generated by the HRIS to make decisions				

Table 2. Self-assessment of DHMT actual practice in Human Resource management

1. Domain	2. Function	3. What is the process involved in conducting this function?	4. Please describe the consequences of the current practice(s)	5. What is your assessment/reflection on the process?	6. Comments
HRH policy	Setting HR policy/regulations locally				
	Monitoring policy/regulation implementation				
HRH planning, recruiting and deploying	Forecasting staffing needs				

	Modifying/adjusting staffing norms according to need				
	Developing job descriptions for staff				
	Hiring and dismissing staff				

	Deploying/posting staff to the health facilities in the district				
HRH financing	Setting salaries/benefits for certain staff categories				
	Mobilising, investing and managing local resources for HR				

	Establishing incentive schemes for staff				
**Performance management and supervision**	Appraising staff's performance				
	Supervising staff				

**Continuing education/in-service training**	Identifying staff capacity needs				
	Organizing in-service training for staff				
**HRH information**	Developing and/or adapting HRH information systems				

	Managing the HRH information system				
	Using data generated by the HRIS to make decisions				
